# Breast Self-Examination: Evaluating Knowledge, Attitudes, and Practices Among Female Medical Students

**DOI:** 10.7759/cureus.86419

**Published:** 2025-06-20

**Authors:** Shruti Raghavan, Shraddha Mishra, Abhijit Das, Sandhya Singh

**Affiliations:** 1 Department of Community Medicine, Government Bundelkhand Medical College, Sagar, IND; 2 Department of Community Medicine, Tripura Medical College and Dr. BRAM Teaching Hospital, Agartala, IND

**Keywords:** attitude, breast self-examination (bse), knowledge, medical students, practice

## Abstract

Background

Breast cancer is the most common cancer among women globally and the leading cause of cancer-related deaths in India. Early detection through breast self-examination (BSE) is crucial for improving prognosis, particularly in resource-limited settings. Despite its straightforward nature, the practice of BSE remains infrequent. This study aimed to assess the knowledge, attitude, and practice of BSE among female medical students in Sagar, Madhya Pradesh, India.

Methods

This cross-sectional observational study was conducted from January to March 2023, involving 146 undergraduate female students at Government Bundelkhand Medical College, Sagar. Data were collected using a pretested, semi-structured, self-administered questionnaire. Knowledge, attitude, and practice scores were categorized based on the mean. The chi-square test was utilized to assess differences in knowledge, attitude, and practice between groups, while Pearson’s correlation was employed to examine the relationships among these three domains.

Results

The mean age of participants was 21.1 ± 1.8 years, with the majority being third-year medical students. Overall, 52.1% (76/146, 95% CI: 43.6-60.4%) demonstrated inadequate knowledge of BSE, whereas only 47.9% (70/146, 95% CI: 39.6-56.4%) exhibited adequate knowledge. Although 68.5% (100/146) of students were aware of BSE, a detailed understanding of its appropriate timing, frequency, and technique was limited. Negative attitudes toward BSE were reported by 51.4% (75/146, 95% CI: 43.0-59.7%), and only 49.3% (72/146, 95% CI: 41.0-57.7%) reported satisfactory BSE practices. Notably, a mere 29.5% (43/146) of participants had ever performed BSE, with only 8.2% (12/146) adhering to the recommended monthly practice. An upward trend in knowledge, attitude, and practice scores was observed with increasing age and academic year. Moreover, there were notable positive correlations identified among knowledge, attitude, and practice.

Conclusions

This study underscores the insufficient knowledge, attitudes, and practices regarding BSE among female medical students in Sagar, Madhya Pradesh. Despite its simplicity and cost-free nature, BSE remains underutilized due to informational gaps and psychological barriers. However, the improvement in knowledge, attitude, and practice with academic progression suggests that structured education and clinical exposure can enhance BSE adoption.

## Introduction

Breast cancer is the most prevalent cancer among women globally, representing approximately one-quarter (23.8%) of all female cancers [1]. According to the Global Cancer Observatory (GCO), the estimated five-year prevalence of breast cancer in women of all ages reached 7,790,717 cases (30.3%), with 685,000 deaths recorded in 2020 [1,2]. In the Southeast Asia region, breast cancer-related deaths are projected to rise by 61.7% by 2040 [1]. Similarly, in India, it is the leading cancer among women, constituting 26.6% of all female cancers [1]. Notably, in Madhya Pradesh, breast cancer accounts for 31% of all cancers in women [3].

Unfortunately, survival rates in India are lower compared to Western countries, attributed to factors such as earlier onset, late-stage diagnosis, delays in initiating definitive treatment, and fragmented care. Screening and early detection are critical for reducing breast cancer mortality, offering less invasive treatment options, and improving overall prognosis. Regular screening and increased awareness enable earlier diagnosis, which is essential for effective treatment and long-term survival [4]. The World Cancer Report 2020 underscores that early detection and timely treatment are the most effective strategies for controlling breast cancer [5]. Early detection hinges on awareness of breast cancer signs and symptoms, as well as the utilization of screening methods such as breast self-examination (BSE), mammography, and clinical breast examination.

Both mammography and clinical breast examinations require hospital visits, which can be a barrier in low-resource settings [6]. In developing countries, BSE is often recommended as it is easy to perform, convenient, private, safe, and requires no special equipment. Studies have shown a positive association between regular BSE and the early detection of breast cancer, with many early tumors being self-discovered. Despite being a simple, quick, and cost-free procedure, the practice of BSE remains low and shows considerable variation across countries [7-15]. In India as well, the uptake of BSE is limited and varies widely across different regions and populations. Several factors have been identified as barriers to regular BSE practice, including lack of time, low self-efficacy in performing the technique correctly, fear of detecting a lump, and feelings of embarrassment associated with breast manipulation [16-20].

Female medical students play a crucial role in promoting BSE through their capacity to educate and influence peers, families, and the broader community. As future healthcare professionals, their knowledge and attitudes significantly impact public awareness and acceptance of BSE. While numerous studies have evaluated knowledge, attitudes, and practices concerning BSE in various countries, including certain regions of India, there is a significant paucity of data from Madhya Pradesh. Consequently, the present study examined the knowledge, attitudes, and practices related to BSE among female undergraduate medical students at a government-run medical college in Sagar, Madhya Pradesh.

This study was previously presented as a poster at the International Conference on Kaleidoscopic Insights into Reproductive and Child Health, organized by ICMR-National Institute for Research in Reproductive and Child Health (ICMR-NIRRCH), Mumbai, on January 24, 2023.

## Materials and methods

Study design, settings, and duration

This was an institution-based observational cross-sectional study conducted in Government Bundelkhand Medical College, Sagar, Madhya Pradesh, between January and March 2023.

Study population

The study population consisted of undergraduate female medical students studying at the Government Bundelkhand Medical College, Sagar, Madhya Pradesh.

Sample size and sampling

The sample size was calculated using the following formula:

\[ n = \frac{Z^2pq}{d^2}, \]

where p = 96%, representing the proportion of students who recognized that BSE is helpful in the early detection of breast cancer [21]. With a 99% confidence interval, 5% precision, and 10% non-response, the minimum required sample size was 115. However, universal sampling was done to include all undergraduate female medical students studying during that period.

Selection criteria

All undergraduate female medical students of Government Bundelkhand Medical College were included, while participants were excluded based on the following criteria: (1) all those who were not willing to participate in the study; (2) those absent on the day of the visit; and (3) any incomplete response.

Recruitment and procedures

The questionnaire was distributed by the study team during a scheduled theory class to all female students present on that day. Participants were given one day to complete the survey and return the forms to the research team. The study targeted a cohort of 165 female medical students, of whom 146 completed the questionnaire, yielding a response rate of 88.5%.

Data collection and analysis

Data were collected using a self-administered, semi-structured, pretested questionnaire comprising four sections. The first section gathered demographic information, including participants’ age and year of MBBS study. The second section assessed knowledge regarding breast cancer and BSE through 15 items, including questions on awareness, sources of information, correct timing, procedure, and warning signs, as well as known risk factors. Each correct response was awarded one point, with a total possible score ranging from 0 to 15; knowledge was categorized as “good” or “poor” based on the mean score. The third section evaluated attitudes toward BSE using a 5-point Likert scale (1 = strongly disagree to 5 = strongly agree) across nine statements, including two negatively worded items that were reverse-scored. Each statement was scored on a 5-point Likert scale (ranging from 1 to 5), and the scores were summed to obtain a total attitude score ranging from 9 to 45. Attitudes were categorized as positive or negative based on the mean total score. The fourth section focused on BSE practices, including frequency and method, and explored reasons for non-practice. Practice scores ranged from 0 to 5, with “good” and “poor” practices similarly classified using the mean. For knowledge, attitude, and practice domains, scores greater than or equal to the mean were classified as “good” or “positive,” while scores below the mean were considered “poor” or “negative.”

Data analysis was conducted using IBM SPSS Statistics for Windows, Version 26.0 (Released 2019; IBM Corp., Armonk, NY, USA). Quantitative data were summarized as mean and standard deviation, while qualitative data were presented as frequency and percentage. The chi-square test was employed to evaluate the differences in knowledge, attitude, and practice between groups. Pearson’s correlation was applied to observe the correlation among these three domains. A p-value less than 0.05 was considered statistically significant.

Ethical consideration

The Institutional Ethics Committee of Government Bundelkhand Medical College, Sagar, approved this study (IECBMC/2023/106). The purpose of the study was explained to the participants, and informed consent was obtained before their participation. No personally identifiable information was collected to ensure anonymity and minimize refusal rates. As this study was conducted over a short duration and involved only baseline data collection, no training intervention was implemented immediately thereafter. However, a general awareness session was conducted at a much later stage.

## Results

The mean age of the participants was 21.1 ± 1.8 years, and the majority of the participants were third-year professional students (Table 1).

**Table 1:** Baseline characteristics of participants (n = 146)

Variables	Subgroup	Frequency	Percentage
Age group (in years)	18-20	54	37
	21-25	92	63
Year of education	First year	36	24.7
	Second year	22	15.1
	Third year	45	30.8
	Final year	43	29.5

**Figure 1:**
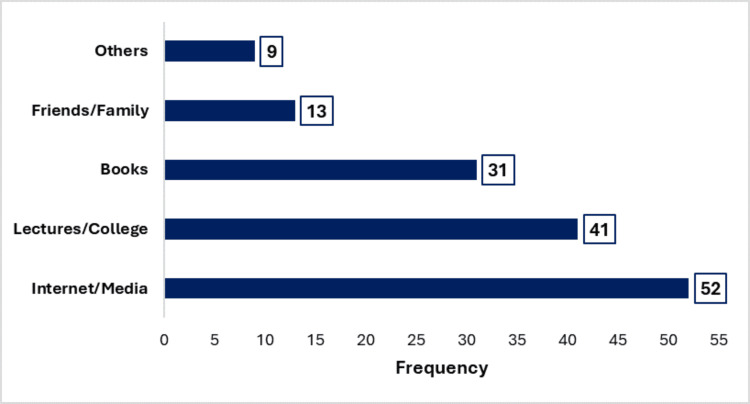
Sources of information among participants Multiple sources were allowed.

More than half of the participants (52.1%, 76/146, 95% CI: 43.6-60.4%) demonstrated poor knowledge regarding BSE. While 70 participants (47.9%, 95% CI: 39.6%-56.4) showed good knowledge. A total of 68.5% (100/146) of the students reported having heard about BSE, with sources of information being diverse (Figure 1). However, knowledge across specific domains of BSE was inconsistent. For instance, 37.0% (54/146) correctly identified that BSE should be performed monthly, and 27.4% (40/146) knew that BSE should be initiated after the onset of menarche. Only 23.3% (34/146) correctly recognized the optimal timing for performing BSE, approximately one week after menstruation. Moreover, 43.8% (64/146) demonstrated correct knowledge of the procedure for conducting BSE.

**Figure 2:**
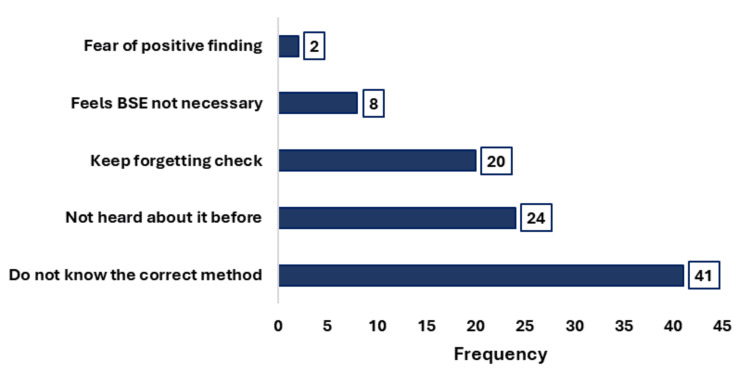
Reasons for not performing BSE Multiple reasons were allowed. BSE: breast self-examination

A negative attitude toward BSE was observed in 75 participants (51.4%, 95% CI: 43.0-59.7), whereas 71 participants (48.6%, 95% CI: 40.3-57.0) exhibited a positive attitude. Overall, 49.3% of participants (72/146; 95% CI: 41.0%-57.7%) demonstrated good practice of BSE, while 50.7% (74/146; 95% CI: 42.3%-59.0%) reported poor practice. Despite this, only 29.5% (43/146) had ever performed BSE, with just 8.2% (12/146) practicing it monthly and 22.6% (33/146) performing it correctly. Among participants who practiced BSE, multiple reasons were reported, with the majority (24.7%, 36/146) performing it as a routine self-check. A smaller proportion cited specific reasons such as noticing a lump (2.1%, 3/146), receiving medical advice (2.1%, 3/146), experiencing pain or discharge (1.4%, 2/146), or having a positive family history of breast cancer (0.7%, 1/146). The most common reason for not practicing BSE was a lack of knowledge about the correct method, followed by limited awareness and forgetfulness (Figure 2).

An analysis of age groups showed that students aged 21-25 years had higher levels of knowledge (53.2%, 49/92), positive attitude (52.1%, 48/92), and good practice (55.4%, 51/92) compared to those aged 18-20 years, though the differences were not statistically significant (Table 2).

**Table 2:** Distribution of knowledge, attitude, and practice across age groups Each cell depicts the frequency and percentage across the age groups.

Domain	Category	Age group (in years)		Chi-square value	p-value
		18-20	21-25		
Knowledge	Poor	33 (61.1%)	43 (46.7%)	2.816	0.093
	Good	21 (38.8%)	49 (53.2%)		
Attitude	Negative	31 (57.4%)	44 (47.8%)	1.25	0.263
	Positive	23 (42.5%)	48 (52.1%)		
Practice	Poor	33 (61.1%)	41 (44.5%)	3.727	0.054
	Good	21 (38.8%)	51 (55.4%)		

There was an increasing trend in BSE-related knowledge, attitude, and practice with advancing years of education (Table 3). The proportion of students with good knowledge rose from 36.1% (13/36) in the first year to 65.1% (28/43) in the final year. Similarly, positive attitude increased from 38.9% (14/36) to 58.1% (25/43), and good practice improved from 33.3% (12/36) in the first year to 62.8% (27/43) in the final year.

**Table 3:** Distribution of knowledge, attitude, and practice across years of education Each cell depicts the frequency and percentage across the years of education.

Domain	Year of education				Chi-square value	p-value
	First	Second	Third	Final		
Knowledge						
Good	13 (36.1%)	9 (40.9%)	20 (44.4%)	28 (65.1%)	7.757	0.051
Poor	23 (63.9%)	13 (59.1%)	25 (55.6%)	15 (34.9%)		
Attitude						
Positive	14 (38.9%)	10 (45.5%)	22 (48.9%)	25 (58.1%)	3.014	0.389
Negative	22 (61.1%)	12 (54.5%)	23 (51.1%)	18 (41.9%)		
Practice						
Good	12 (33.3%)	10 (45.5%)	23 (51.1%)	27 (62.8%)	6.992	0.072
Poor	24 (66.7%)	12 (54.5%)	22 (48.9%)	16 (37.2%)		

Pearson correlation analysis revealed statistically significant positive relationships among knowledge, attitude, and practice regarding BSE (Table 4). Knowledge was positively correlated with attitude (r = 0.246, p = 0.003) and practice (r = 0.287, p < 0.001). Similarly, attitude showed a significant positive correlation with practice (r = 0.383, p < 0.001). These findings suggest that higher knowledge and more favorable attitudes are associated with better BSE practices.

**Table 4:** Correlation between knowledge, attitude, and practice scores ^**^ p-value <0.01 level

Variables	Knowledge	Attitude	Practice
Knowledge	1	0.246^**^	0.287^**^
Attitude	-	1	0.383^**^
Practice	-	-	1

## Discussion

This research on BSE knowledge, attitudes, and practices among female medical students in Madhya Pradesh, India, provides valuable insights into an important public health issue. The study identified substantial knowledge deficits regarding BSE among participants, with more than half demonstrating inadequate awareness. However, a study conducted in South India reported a comparatively higher level of knowledge regarding BSE [21]. Although only a minority demonstrated good knowledge, the findings underscore a significant gap in breast cancer preventive practices. In our study, the majority of participants reported having heard about BSE, with information sources ranging from online platforms and media to academic lectures, books, and peer discussions. Similarly, previous studies by Alshafie et al. and Kawalkar and Koparkar reported high levels of BSE awareness among students [14,22]. Notably, lectures and healthcare professionals have been consistently identified as primary sources of BSE knowledge, although media and peer networks also contribute substantially [14,22,23]. Knowledge across specific domains of BSE was found to be inconsistent. While some participants were aware of the recommended frequency and timing of BSE, such as performing it monthly and around one week after menstruation, many lacked accurate information. Awareness of the appropriate age to initiate BSE and understanding of the correct technique, including visual inspection in front of a mirror, were also limited among respondents. These findings are consistent with previous studies from India, such as that by Nair and Gopinath, which similarly reported partial knowledge across various aspects of BSE practice [21].

A positive attitude toward BSE was observed in less than half of the participants, while 51.4% (75/146) exhibited a negative attitude. Similarly, Alshafie et al. reported that just over half of the students demonstrated a favorable attitude toward BSE [14]. Attitudes toward anything are often shaped by underlying knowledge, and evidence suggests that educational interventions can significantly influence these perceptions [24]. For instance, the study by Ranganath et al. demonstrated a marked improvement in students’ attitudes following a structured training program, as reflected in enhanced post-intervention scores [23]. This underscores the importance of targeted educational efforts in promoting positive health behaviors. Approximately half of the participants in the present study demonstrated poor practices related to BSE. Only a small proportion had ever performed BSE, and even fewer adhered to the recommended monthly frequency. This is notably lower than findings from previous studies, such as those by Kawalwar et al. and Alshafie et al., which reported higher rates of BSE practice among participants [14,22]. Among those who had performed BSE in our study, the most common reason cited was routine self-checking. Other reported motivations included the presence of symptoms such as a lump, medical advice, pain or discharge, and a positive family history of breast cancer, though these were less frequently mentioned.

Barriers to practicing BSE were also evident. The predominant reason was a lack of knowledge regarding the correct method, followed by limited awareness and forgetfulness. These findings are in line with previous research by Kawalkar and Koparkar, who identified additional barriers such as feelings of awkwardness, time constraints, and a perceived lack of necessity. Furthermore, among those who practiced BSE, irregularity was common, with many lacking a fixed schedule and only a minority performing it monthly as recommended [22]. Evidence from previous studies suggests that structured training and educational interventions can significantly enhance both the frequency and consistency of BSE practice [23]. These findings emphasize the need for targeted educational interventions to improve both knowledge and consistent practice of BSE. An encouraging finding of the study was the progressive improvement in knowledge, attitudes, and practices related to BSE with advancing years of medical education. This trend reflects the positive influence of academic exposure and training on students' understanding and engagement with BSE. The study also identified significant positive correlations among knowledge, attitudes, and practices, indicating that increased knowledge is associated with more favorable attitudes and better practices. Such interrelationships have been consistently documented in previous literature, underscoring the importance of integrated educational strategies to promote comprehensive awareness and adoption of preventive health behaviors [14,20].

Our study provides contextual relevance by addressing BSE within Madhya Pradesh, India, contributing to an underexplored area. However, several limitations exist. The single-institution focus may limit generalizability to broader populations. The use of self-reported data introduces potential response bias. The cross-sectional design further limits the ability to infer causal relationships or assess changes over time. The absence of qualitative data restricts deeper insights into participants’ attitudes and perceived barriers, while limited consideration of cultural and societal influences may overlook important contextual determinants affecting BSE-related behaviors.

## Conclusions

Our study evaluates the knowledge, attitude, and practice of BSE among female medical students in Sagar, India. Despite being a simple, cost-free detection method, BSE remains underutilized among future healthcare providers. Most respondents lacked adequate knowledge, held negative attitudes, or did not practice BSE regularly. Low knowledge about timing, technique, and initiation age indicates gaps in the medical curriculum. Negative attitudes from fear and lack of confidence highlight the need for addressing psychological barriers. BSE-related knowledge, attitude, and practice scores improved with advancing academic years, showing that clinical exposure enhances awareness. Given their future role, empowering medical students with BSE knowledge is essential. Institutional commitment to preventive health education can reduce the breast cancer burden in India.

## Appendices

**Table 5:** Study questionnaire

Breast Self-Examination: Evaluating Knowledge, Attitudes, and Practices Among Female Medical Students						
Section 1: Basic information						
Age (in years)						
MBBS Year	First	Second	Third	Fourth	-	-
Section 2: Knowledge regarding breast self-examination (BSE) & breast cancer						
Have you heard of BSE before?	Yes	No	-	-	-	-
If yes, what is your source of awareness? (multiple options can be selected)	Friends/family	Lecture/college	Books	TV	Internet	Other
How often should BSE be performed?	Weekly	Monthly	Yearly	I Don't Know	-	-
What is the correct time to initiate BSE?	After Attaining Menarche	Above 18 Years	At Beginning of Teenage Years	I Don't Know	-	-
What is the appropriate time to perform BSE?	A week before menstruation	A week after menstruation	During menstruation	Anytime	I don't know	-
Is BSE important in early detection of breast cancer?	Yes	No	Don't know	-	-	-
What is the appropriate place to perform BSE?	In front of a mirror	Supine position	I don't know	-	-	-
How is breast self-examination done?	Palpation using palm & three finger pulps	Palpation with one finger	I don’t know	-	-	-
Are changes in the shape and color of the breast the signs of breast cancer?	Yes	No	-	-	-	-
Are lumps in the breast and around the armpit the signs of breast cancer?	Yes	No	-	-	-	-
Are nipple discharge and retraction the signs of breast cancer?	Yes	No	-	-	-	-
Is a positive family history of breast cancer a risk factor for developing breast cancer?	Yes	No	-	-	-	-
Is increasing age a risk factor for developing breast cancer?	Yes	No	-	-	-	-
Are early menarche and late menopause risk factors for developing breast cancer?	Yes	No	-	-	-	-
Is obesity a risk factor for developing breast cancer?	Yes	No	-	-	-	-
Are taking oral contraceptive pills and polycystic ovary syndrome risk factors for developing breast cancer?	Yes	No	-	-	-	-
Section 3: Attitude section (mark the most appropriate one)						
Performing BSE makes me feel unpleasant.	Strongly disagree	Disagree	Neutral	Agree	Strongly agree	-
All women should perform BSE.	Strongly disagree	Disagree	Neutral	Agree	Strongly agree	-
I am afraid to think about CA breast, so I avoid BSE.	Strongly disagree	Disagree	Neutral	Agree	Strongly agree	-
I am interested in performing BSE.	Strongly disagree	Disagree	Neutral	Agree	Strongly agree	-
If there is a lump, I prefer to seek medical help immediately.	Strongly disagree	Disagree	Neutral	Agree	Strongly agree	-
I am keen to search for information regarding BSE.	Strongly disagree	Disagree	Neutral	Agree	Strongly agree	-
I am comfortable discussing about BSE with my friends and family.	Strongly disagree	Disagree	Neutral	Agree	Strongly agree	-
I am willing to learn correct BSE techniques through workshops.	Strongly disagree	Disagree	Neutral	Agree	Strongly agree	-
I think BSE is a good practice.	Strongly disagree	Disagree	Neutral	Agree	Strongly agree	-
Section 4: Practice regarding BSE						
Do you perform BSE?	Yes	No				
If yes, how often	Weekly	Monthly	Quarterly	Every 6 months	Yearly	Other (specify)
If you performed BSE on yourself, what was your purpose?	For a medical reason	I noticed a lump	Positive family history	Routine self-checkup	Pain or discharge	Other (specify)
How do you perform BSE on yourself?	Palpate with one finger	Palpate with palm and three fingers	I don’t know	Other (specify)	-	-
If No, what is the reasons?	I have fear of positive findings	I forget to do it	I don't know the correct method	I am not interested	I feel it is not necessary	I have not heard about it before
If you identify an abnormality, what will you do?	Tell friends or family	Seek medical help and get tests done	Do nothing about it	Self-medicate	-	-
Do you frequently search for information regarding BSE to be updated?	Yes	No	-	-	-	-
